# “To Fix a Broken Heart”: An Unusual Case of Infective Endocarditis Involving the Mitral Valve With Perforation and Hemodynamic Instability

**DOI:** 10.7759/cureus.18367

**Published:** 2021-09-28

**Authors:** Rezwan Munshi, James R Pellegrini, Allen R Tsiyer, Megan Barber, Ofek Hai

**Affiliations:** 1 Internal Medicine, Nassau University Medical Center, East Meadow, USA; 2 Cardiology, Nassau University Medical Center, East Meadow, USA

**Keywords:** endocarditis, mitral valve endocarditis, staphylococcus aureus, mitral valve perforation

## Abstract

Infective endocarditis (IE), commonly caused by *Staphylococcus aureus*, can affect multiple cardiac structures and lead to significant morbidity and mortality. We present a case of IE with extensive mitral valve involvement causing perforation and hemodynamic compromise.

A 66-year-old Caucasian female presented to the emergency department for progressive altered mental status and lethargy. The patient and family denied history of intravenous drug use (IVDU) on interview. Physical exam revealed tachypnea, tachycardia, lethargy, and fluctuance in the right antecubital fossa draining serous fluid. Initial studies revealed a urinary tract infection, patchy bilateral opacities on chest x-ray, hypoxic respiratory failure, elevated lactate and cardiac markers, leukocytosis, and positive urine toxicology for opioid and benzodiazepine. She was intubated and admitted to the ICU, and later developed acute respiratory distress syndrome with requirement for vasopressors. Antibiotics were started, and blood cultures ultimately grew methicillin-sensitive *S. aureus*. Coronavirus disease 2019 (COVID-19) results were negative. Cardiology was consulted for elevated cardiac markers that were due to myocardial injury in the setting of septic shock. A transthoracic echocardiogram showed a large mobile mass on the anterior mitral leaflet. Further evaluation with transesophageal echocardiogram revealed a large, mobile, and centrally necrotic vegetation on the medial portion of the mitral annulus extending to both the anterior and posterior leaflets. Doppler of the valve showed holosystolic retrograde ejection into the left atrium confirming a perforation. The patient was transferred urgently to a cardiothoracic surgery capable center for operative intervention on the mitral valve.

IE is most commonly caused by *S. aureus *and seen in highest rates among patients with a prosthetic valve, congenital heart disease, and intracardiac device. However, roughly 50% of IE occurs in patients without any valvular disease. Other risk factors include IVDU, valvular disease, and prior history of endocarditis. Clinical diagnosis of IE is made using the Duke’s criteria, with echocardiogram and bacteremia playing a major role. The initial management involves empiric antibiotics until a pathogen is identified. Surgical consult is also suggested, and indications for surgery include heart failure due to valve dysfunction, uncontrolled infection, prevention of embolism, and hemodynamic compromise. Prompt recognition and intervention is crucial in the prevention of mortality in patients with IE leading to mitral perforation and hemodynamic compromise.

## Introduction

Infective endocarditis (IE) is caused by an infection in the bloodstream that travels to the heart and infiltrates coronary tissue. The most common causative agent is *Staphylococcus aureus *[1]. It is most common among patients with underlying valvular disease, prosthetic valves, congenital coronary anomalies, intracardiac devices, and intravenous drug use (IVDU). Our case highlights a less frequently encountered IE in a patient with suspected IVDU and extensive mitral valve involvement causing perforation and valvular insufficiency.

## Case presentation

A previously well 66-year-old Caucasian female with a history of hypothyroidism and depression presented to the emergency department (ED) for altered mental status and lethargy noticed by her son. She had become progressively lethargic over the three days prior to presenting to the hospital. Vitals in the ED were stable. Although the patient and family history were non-congruent with intravenous drug use (IVDU), the patient had track marks and an abscess present in the antecubital fossa. Physical exam revealed tachypnea, lethargy, and an abscess in the right antecubital fossa draining serous fluid. The initial evaluation revealed a urinalysis consistent with a urinary tract infection, patchy opacities in the lungs on chest x-ray that was greater on the right likely representative of atypical pneumonia or pulmonary edema, arterial blood gas revealing respiratory alkalosis with hypoxia, elevated cardiac markers, complete blood count showing leukocytosis, and urine toxicology positive for benzodiazepine and opioid. She was intubated and admitted to the medical ICU (MICU) for acute hypoxic respiratory failure secondary to benzodiazepine and opioid overdose, which later progressed to acute respiratory distress syndrome. Propofol and Ativan drips were used as sedation for improved ventilator synchronization. Though initially her chest x-ray findings and respiratory decompensation were thought to be due to coronavirus disease 2019 (COVID-19), her test results for COVID-19 were negative.

Upon arriving to the MICU, she was found to have hypotension with tachycardia, and along with leukocytosis and multiple sources of infection, she was diagnosed with septic shock requiring vasopressors. Broad-spectrum antibiotics were empirically started given the various sources of infection. Blood cultures originally grew gram-positive cocci in clusters that were later identified as methicillin-sensitive *S. aureus* (MSSA).

**Figure 1:**
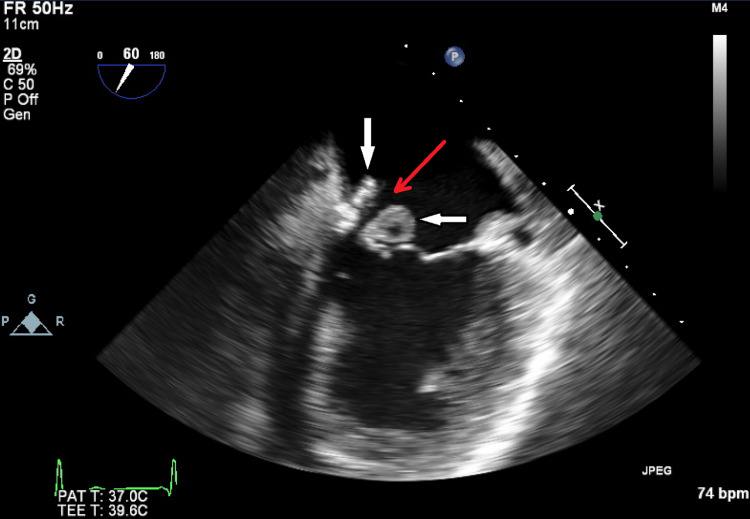
Transesophageal echocardiogram with a mid-position view displaying a vegetation (white arrows) on the mitral annulus with extension to the anterior and posterior mitral leaflets and perforation of the anterior mitral leaflet (red arrow)

**Figure 2:**
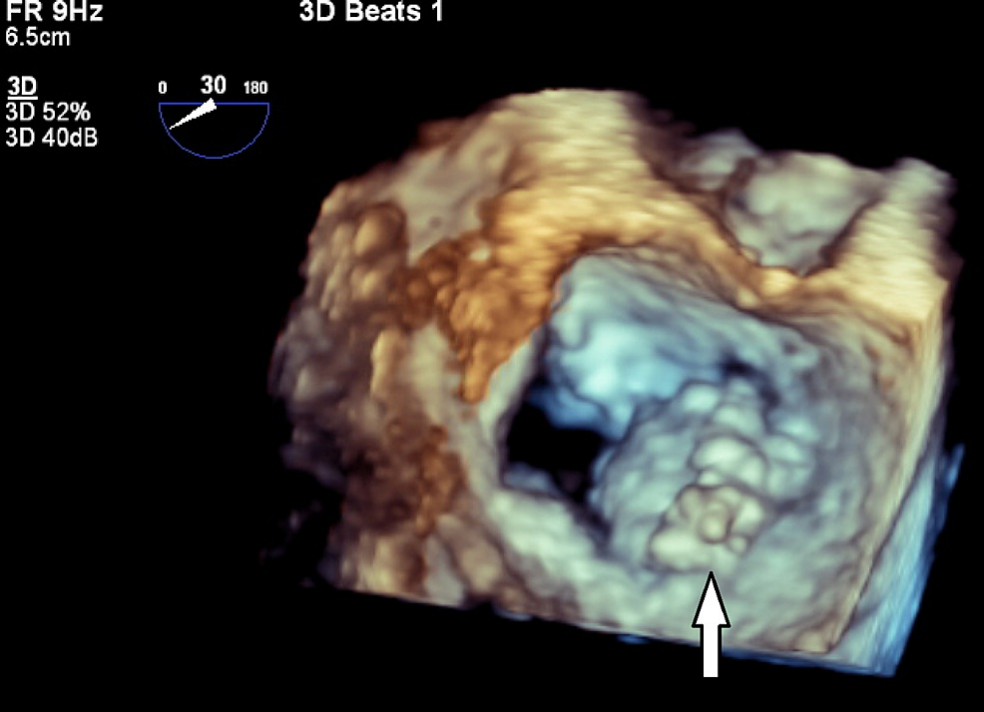
Transesophageal echocardiogram with a three-dimensional (surgeon’s view) image displaying a vegetation (white arrow) on the mitral annulus

**Figure 3:**
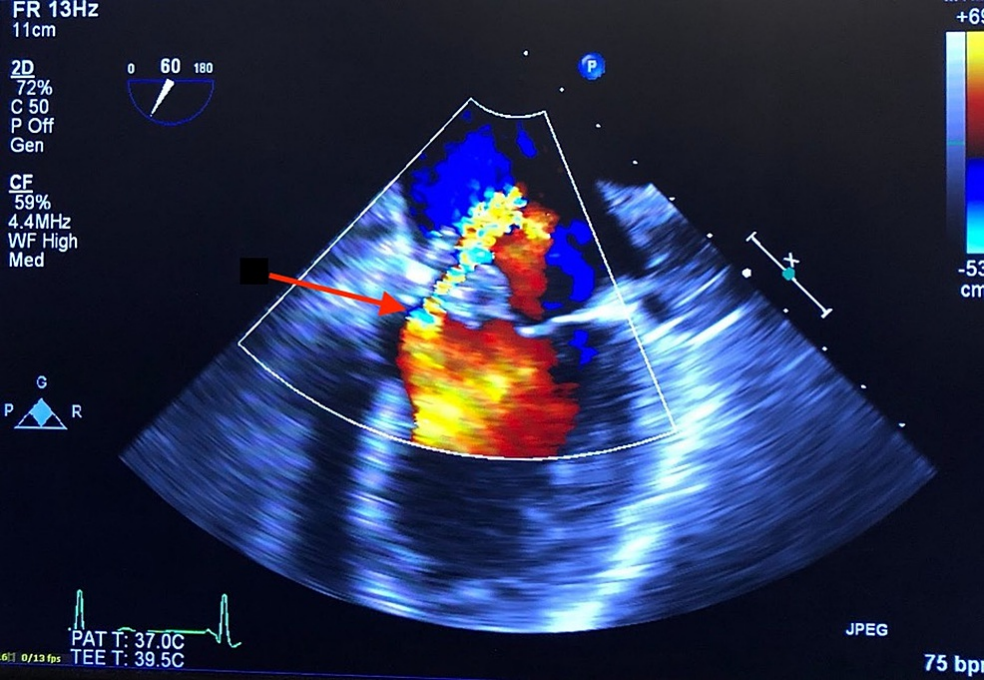
Transesophageal echocardiogram with a mid-position view showing severe mitral regurgitation (red arrow) going through the necrotic core of the vegetation

Cardiology was consulted for elevated cardiac markers, which continued to trend upwards until troponin leveled off at 11.70 ng/mL, attributable to type 2 non-ST elevation myocardial infarction in the setting of septic shock and tachycardia. A transthoracic echocardiogram (TTE) was obtained for the patient, which showed a large and mobile vegetation on the mitral annulus (Figure 1) extending to the anterior and posterior mitral leaflets with perforation, along with moderate to severe mitral regurgitation and a hyperdynamic global left ventricular function. Further study with a transesophageal echocardiogram (TEE) showed a large pedunculated, mobile, and centrally necrotic vegetation on the medial portion of the mitral annulus (Figure 2), as well as severe mitral regurgitation through the necrotic core of the vegetation (Figure 3). No evidence of tricuspid valve involvement was present on imaging. The patient was medically optimized and transferred urgently to a center with cardiothoracic surgery capabilities for operative intervention on the mitral valve regurgitation. The patient received left heart catheterization and coronary angiography that showed mild luminal coronary artery disease without any obstruction and an elevated left ventricular end diastolic pressure of 17 mmHg. Surgical confirmation of the infected valve was made and the patient then proceeded to receive extensive debridement of the mitral annulus and a bioprosthetic mitral valve replacement. Intra-operative TEE showed no paravalvular leak. After the surgery, computed tomography of the head did not show any evidence of mycotic aneurysm. The patient underwent a TTE during a follow-up visit after discharge that showed a bioprosthetic mitral valve with normal function and no other abnormalities.

## Discussion

*S. aureus* is the most common organism in the industrialized world for infections, and it is the most prevalent organism leading to IE [1,2]. In the United States, *S. aureus* bacteremia has an annual incidence of 38.2-47.7 per 100,000 [3]. The most common risk factors for *S. aureus* endocarditis are valvular disease, community acquisition, IVDU, congenital cardiac anomalies, intracardiac devices, and prior endocarditis [1,4].

*S. aureus* bacteremia is commonly associated with a right-sided IE, and this is most commonly seen with IVDU [5]. The most frequently involved valve is the tricuspid valve in IVDU. Furthermore, only 20%-30% of IE in IVDU affects the aortic or mitral valves, and 5%-10% involves multiple valves. In IE, the predilection for right-sided valves in IVDU can be due to the fact that microbial surface components recognizing adhesive matrix molecules have greater expression on the right side [5]. *S. aureus* IE has been shown to increase the risk of complications such as embolic disease and abscess formation in addition to a higher mortality risk [6].

A vegetation is mainly a collection of fibrin, platelets, and bacteria. The formation of a vegetation initially involves an insult to the valve such as turbulent blood flow, direct injury from an intravenous catheter, or debris from IVDU [7]. This leads to a thrombus formation that allows bacteria to adhere to the damaged endocardium and form a biofilm containing polysaccharide and proteinaceous matrix, allowing it to persist and resist antibiotic penetration [7,8]. The site of the insult is directed by the Venturi effect, in which deposition of the thrombus occurs on the lower pressure side. In the case of IE on the mitral valve, the insult would occur on the atrial side of the leaflets [7].

Clinical diagnosis of IE is made using the Duke’s criteria, which consist of major and minor criteria. Major criteria include sustained bacteremia by an organism known to cause endocarditis, endocardial involvement (echocardiogram showing vegetation, abscess, perforation, prosthetic dehiscence) or a new valvular regurgitation. Minor criteria consist of any predisposing condition, fever, vascular phenomena (Janeway lesions, intracranial hypertension, pulmonary embolism, mycotic aneurysms), immune phenomena (Roth spots, Osler nodes, glomerulonephritis, rheumatoid factor), positive blood cultures, or positive TTE or TEE findings (not meeting major requirement). A clinical diagnosis of IE is established when two major, one major and three minor, or five minor criteria are met [9]. In the clinical setting, a patient presenting with acute IE will typically have an abrupt onset of fever and rigors. Once clinical suspicion is high, a full workup should follow that includes echocardiogram, three sets of blood cultures, chest x-ray, urinalysis, and rheumatoid factor [7]. In the case we present, our patient met two of the major criteria with endocardial involvement shown on echocardiogram and bacteremia with an organism known to cause endocarditis, leading us to a pre-operative diagnosis of IE.

On echocardiogram, any of the following findings are diagnostic: oscillating intracardiac mass that is on either a valve or in line with a regurgitant jet or on an implanted material, abscess, or particle dehiscence of a prosthetic valve [10]. In our case, the echocardiogram demonstrated a large, pedunculated, and mobile vegetation measuring 20 x 8 mm on the anterior mitral leaflet. This was associated with severe eccentric mitral regurgitation through the necrotic core of the vegetation. Nonetheless, the clinical picture should be taken as a whole, utilizing imaging and clinical findings to avoid false positives and negatives. Furthermore, not only should an echocardiogram be used for diagnosis, but also it should be repeated to monitor therapeutic response and post-surgical repair [11]. A TEE is preferred to a TTE as it offers greater resolution due to the higher frequency used and less acoustic shadowing from prosthetic valves. It also has the ability to detect structures that are smaller in diameter and better visualize perforations as compared to a TTE [5]. Leaflet perforation can be a result of the extension of necrosis [11]. It can be defined on an echocardiogram using Doppler as shunting of a high-velocity jet of blood [11,12]. A finding of leaflet perforation is on the more severe spectrum of IE [11].

Previous IE, aortic involvement, and New York Heart Association functional class III-IV (hemodynamic compromise) are risk factors associated with an increased risk of valvular perforation [12]. Of particular significance is previous IE as patients have been found to have perforations at the site of previous infection. The absence of these risk factors in our case should encourage physicians to maintain a high level of suspicion. In addition, the severity of the defect can determine whether the dysfunction is great enough to lead to hemodynamic compromise. Perforation at the aortic valve was correlated to a worse prognosis compared to mitral valve perforation due to the higher risk of hemodynamic instability [12]. Regardless, one should stay vigilant when mitral valve involvement is confirmed due to the risk of clinical deterioration in such patients.

Patients diagnosed with IE should receive empiric antibiotics until a pathogen has been determined via blood cultures. In most cases, MSSA is the pathogen and is typically treated with a beta-lactam agent, with or without gentamicin [13]. In those who have a hypersensitivity to penicillin, cefazolin serves as a worthy alternative. In patients with an anaphylactic penicillin allergy, it is appropriate to start vancomycin as the sole agent [7]. Blood cultures should be drawn every 24-48 hours after the initiation of antibiotics and continued until they are negative [7]. A surgical consultation is suggested for any patient who presents with IE. The above was followed in our case; however, the patient had to be stabilized prior to the transfer to a facility with cardiothoracic surgery capabilities. There are several predictors of a course that may require surgical intervention, although there are three principal indications: heart failure due to valve dysfunction, uncontrolled infection, and prevention of embolism [14]. Some other features are large-diameter (>10 mm) vegetations, embolic events, severe valvular insufficiency, abscess cavities/pseudoaneurysms, perforation, dehiscence/rupture/fistula, or heart failure not amenable to medical treatment [2]. Surgical intervention is required when there is a hemodynamic compromise, as found in our patient. In such patients, intervention within the first seven days is associated with a lower mortality compared to conservative medical management [15].

Current recommendations for IVDU include referral to a program for assistance to stop using IV drugs [2]. Patients have to be educated about signs and symptoms (i.e. fever, chills, distress) of IE in order to seek medical care immediately if there is a potential recurrence. In addition, patients should be monitored for the progression of heart failure or delayed side effects from the antibiotic regimen [2]. The long-term follow-up (several months to years) after initial treatment should include blood cultures in addition to precautions, to be followed, given during the short-term follow-up [2]. It is also of great importance to stress dental hygiene and complete a dental evaluation.

## Conclusions

*S. aureus* bacteremia continues to be an ongoing problem throughout the United States. IE is one of the many and lethal complications of MSSA. It is diagnosed by combining clinical manifestations, blood cultures, and echocardiography. Our case highlights an unusual form of IE in a presumed IVDU with no prior history of valvular dysfunction, where the mitral valve was compromised leading to hemodynamic instability. It is important to recognize rare forms of IE to prevent mortality in high-risk patients.

## References

[1] Chang FY, MacDonald BB, Peacock JE Jr (2003). A prospective multicenter study of Staphylococcus aureus bacteremia: incidence of endocarditis, risk factors for mortality, and clinical impact of methicillin resistance. Medicine (Baltimore).

[2] Baddour LM, Wilson WR, Bayer AS (2015). Infective endocarditis in adults: diagnosis, antimicrobial therapy, and management of complications: a scientific statement for healthcare professionals from the American Heart Association. Circulation.

[3] El Atrouni WI, Knoll BM, Lahr BD, Eckel-Passow JE, Sia IG, Baddour LM (2009). Temporal trends in the incidence of Staphylococcus aureus bacteremia in Olmsted County, Minnesota, 1998 to 2005: a population-based study. Clin Infect Dis.

[4] Rajani R, Klein JL (2020). Infective endocarditis: a contemporary update. Clin Med (Lond).

[5] Frontera JA, Gradon JD (2000). Right-side endocarditis in injection drug users: review of proposed mechanisms of pathogenesis. Clin Infect Dis.

[6] Tintinalli JE, Stapczynski JS, Ma OJ, Yealy DM, Meckler GD, Cline DM (2016). Tintinalli's Emergency Medicine: A Comprehensive Study Guide. https://accessmedicine.mhmedical.com/book.aspx?bookid=2353.

[7] McDonald JR (2009). Acute infective endocarditis. Infect Dis Clin North Am.

[8] Flemming HC, Wingender J (2010). The biofilm matrix. Nat Rev Microbiol.

[9] Durack DT, Lukes AS, Bright DK, Duke Endocarditis Service (1994). New criteria for diagnosis of infective endocarditis: utilization of specific echocardiographic findings. Am J Med.

[10] Li JS, Sexton DJ, Mick N (2000). Proposed modifications to the Duke criteria for the diagnosis of infective endocarditis. Clin Infect Dis.

[11] Sachdev M, Peterson GE, Jollis JG (2002). Imaging techniques for diagnosis of infective endocarditis. Infect Dis Clin North Am.

[12] De Castro S, d'Amati G, Cartoni D (1997). Valvular perforation in left-sided infective endocarditis: a prospective echocardiographic evaluation and clinical outcome. Am Heart J.

[13] Falagas ME, Matthaiou DK, Bliziotis IA (2006). The role of aminoglycosides in combination with a β-lactam for the treatment of bacterial endocarditis: a meta-analysis of comparative trials. J Antimicrob Chemother.

[14] Cahill TJ, Prendergast BD (2016). Infective endocarditis. Lancet.

[15] Jamil M, Sultan I, Gleason TG, Navid F, Fallert MA, Suffoletto MS, Kilic A (2019). Infective endocarditis: trends, surgical outcomes, and controversies. J Thorac Dis.

